# Prosthesis design and placement in reverse total shoulder arthroplasty

**DOI:** 10.1186/s13018-015-0244-2

**Published:** 2015-07-02

**Authors:** David C Ackland, Minoo Patel, David Knox

**Affiliations:** Department of Mechanical Engineering, University of Melbourne, Parkville, Victoria 3010 Australia; Epworth Healthcare, Richmond, Victoria 3121 Australia

**Keywords:** Prosthesis, Biomechanics, Arthropathy, Moment arm, Deltoid, Rotator cuff, Surgery

## Abstract

The management of irreparable rotator cuff tears associated with osteoarthritis of the glenohumeral joint has long been challenging. Reverse total shoulder arthroplasty (RSA) was designed to provide pain relief and improve shoulder function in patients with severe rotator cuff tear arthropathy. While this procedure has been known to reduce pain, improve strength and increase range of motion in shoulder elevation, scapular notching, rotation deficiency, early implant loosening and dislocation have attributed to complication rates as high as 62 %. Patient selection, surgical approach and post-operative management are factors vital to successful outcome of RSA, with implant design and component positioning having a significant influence on the ability of the shoulder muscles to elevate, axially rotate and stabilise the humerus. Clinical and biomechanical studies have revealed that component design and placement affects the location of the joint centre of rotation and therefore the force-generating capacity of the muscles and overall joint mobility and stability. Furthermore, surgical technique has also been shown to have an important influence on clinical outcome of RSA, as it can affect intra-operative joint exposure as well as post-operative muscle function. This review discusses the behaviour of the shoulder after RSA and the influence of implant design, component positioning and surgical technique on post-operative joint function and clinical outcome.

## Introduction

**Fig. 1:**
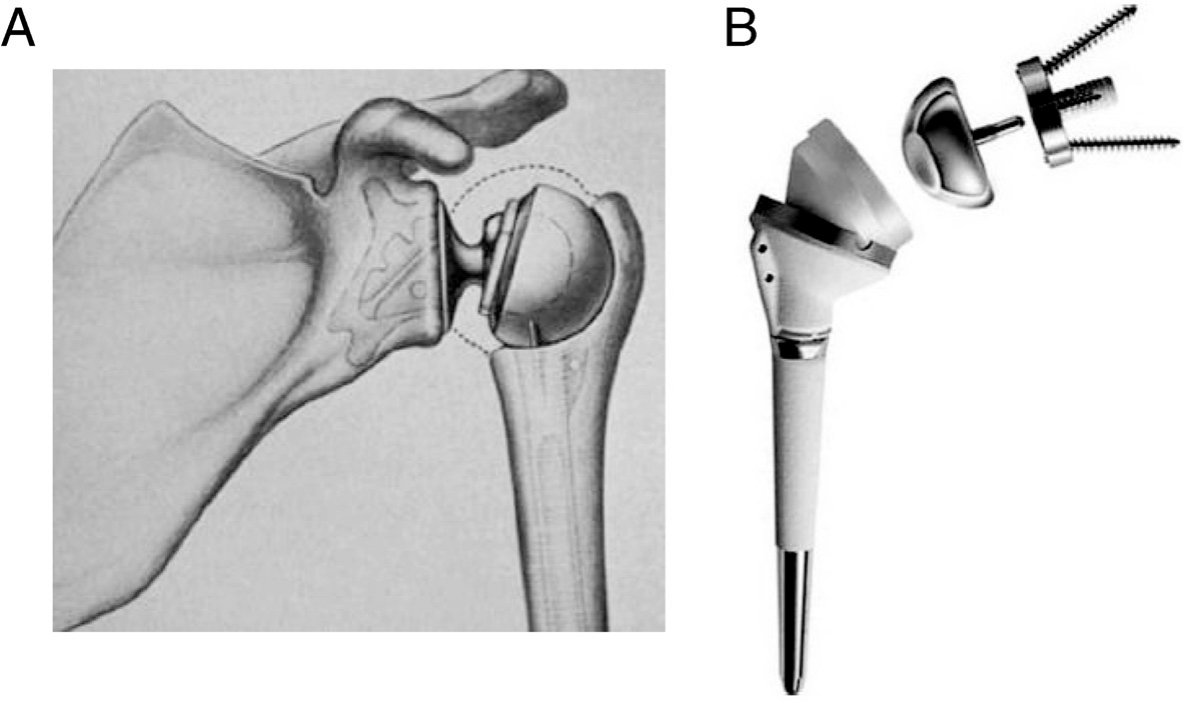
Neer’s constrained reverse shoulder prosthesis concept (**a**) and the Delta III reverse shoulder prosthesis based on Grammont’s original design (**b**)

Reverse total shoulder arthroplasty (RSA) was first described by Grammont et al. in 1987, as a treatment for patients with cuff tear arthropathy for which non-operative treatment options had failed [1]. It involved reversing the polarity of ‘the ball and the socket’ by placing a ‘ball’ component at the glenoid and an articular ‘socket’ at the proximal humerus. Developed over two decades, the Delta III reverse prosthesis was introduced in 1991, and is a direct descendant of the initial Grammont prosthesis (Fig. 1) [2–4]. It has propagated a new family of reverse shoulder implants which are now available from numerous different manufacturers. With improvements in modern implant design and instrumentation, surgical techniques for RSA continue to evolve, as do the surgical indications [5, 6].

While rotator cuff tear arthropathy remains the primary indication for RSA, applications now include a variety of conditions associated with rotator cuff deficiency or dysfunction. These include cuff tear pseudo-paralysis, tumour resection, revision shoulder arthroplasty [1, 5, 7–10], fracture sequelae [10–12] and, lately, severely comminuted non-reconstructable proximal humerus fractures [13]. Complication rates for RSA are as high as 68 % [14], with substantially higher complication rates observed in revision surgery [15, 16]. The most common complications observed in RSA are scapular notching, glenohumeral dislocation, component loosening, acromion or spine of scapula facture, infection, nerve injury and deltoid weakness [17]. With reported complication rates associated with RSA higher than those of conventional anatomic replacement [2, 18–20], significant efforts have been made to refine surgical implantation method and prosthesis design. Variables such as neck-shaft angle of the humerus, glenosphere diameter, eccentricity and lateral offset, glenoid base plate tilt and component fixation are known to influence clinical outcome and can vary significantly in different implant designs and surgical approaches [21].

### Reverse shoulder prosthesis rationale and biomechanics

In the natural shoulder, the rotator cuff actively stabilises the glenohumeral joint by compressing the humeral head against the glenoid [22–24]. This is primarily facilitated by a transverse-plane force couple generated by the simultaneous activity of internal rotators (subscapularis, latissimus dorsi) and external rotators (infraspinatus and teres minor). An important function of this force couple is to resist the upward shear force generated by the deltoid, especially during initiation of abduction [25]. In the case of rotator cuff dysfunction, the ability of the musculature to generate concavity compression may be compromised causing the humeral head to translate superiorly under the superior shear force produced by the deltoid. This may eventually result in acetabularisation of the glenoid and acromion arc and superior glenoid wear [26, 27]. Hemiarthroplasty has been an important standard of care in this environment, but offers only ‘limited goals’ for post-operative function [28–32], with pain relief and range of movement unpredictable [33–35]. Constrained prostheses were introduced to exceed these limited goals with little success. While many such designs may have provided effective short-term pain relief, they were not able to withstand the large shear forces transmitted through the upper limb and typically failed at the glenoid-prosthesis interface [17, 31].

**Fig. 2:**
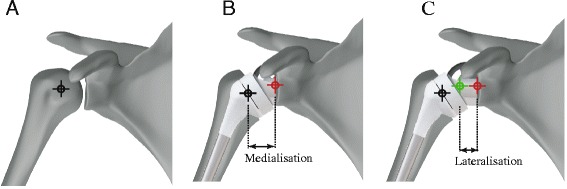
Diagram illustrating joint centre of rotation location for the anatomical shoulder (**a**), reverse shoulder (**b**) and reverse shoulder with a lateral-offset glenoid component (**c**). Medialisation after reverse total shoulder arthroplasty is shown, as well as lateralisation due to a lateral-offset glenoid component. *Black*, *red* and *green bull’s-eyes* indicate joint centre of rotation position for the anatomical shoulder, reverse shoulder and reverse shoulder with a lateral-offset glenoid component, respectively

The Grammont reverse shoulder prosthesis is a semi-constrained implant design. It features a polyethylene humeral cup and a polished cobalt-chromium-molybdenum hemispherical glenoid component (glenosphere). The positioning and geometry of the glenoid component results in a joint centre of rotation located at the glenoid-bone-prosthesis interface. It has been reported that the reverse shoulder prosthesis design shifts the joint centre of rotation medially by up to 20.9 mm, relative to the anatomical shoulder [36] (Fig. 2a, b). This change in geometry of the shoulder joint has four significant mechanical consequences.

Firstly, the humeral cup, oriented at approximately 155° with respect to the long axis of the humerus, covers less than half of the glenosphere [2]. This has the advantage of lowering the humerus, resulting in increased tensioning of the deltoid. However, while greater passive tension in the deltoid may improve deltoid force-generating capacity and joint range of motion, overtensioning of the deltoid may result in fracture of the acromion and reduced shoulder function [37, 38]. Prolonged deltoid overtensioning is also thought to be the cause of mid- to long-term decline in deltoid function.

Secondly, medialisation of the centre of rotation of the glenohumeral joint recruits more fibres of the deltoid during elevation, improving force production and enhancing range of shoulder motion [2]. Thirdly, the glenosphere offers a greater potential arc of movement of the humerus before impingement of the humeral component occurs. Due to the location of the glenohumeral centre of rotation at the glenoid surface, it reduces torque and shear force generated at the glenosphere-bone interface [10], which is a risk factor for base-plate failure in lateralised glenosphere designs.

Finally, RSA results in substantial changes in the moment arms of the muscles spanning the glenohumeral joint [39, 40]. Specifically, the average abduction and flexion moment arms of the middle deltoid have been shown to be 17.2 and 14.8 mm larger after RSA, respectively, with the posterior deltoid also recruited as an abductor (Table 1) [36]. Increased leverage of the deltoid ultimately reduces muscle effort during activities such as lifting and pushing; however, RSA has been shown to decrease the external rotation moment arms of the deltoid and increase the moment arms of the internal rotators [41]. As a consequence, RSA may result in reduced or absent external rotation function, particularly if the infraspinatus and teres minor are damaged.

**Table 1:** Maximum and minimum moment arms of the middle, anterior and posterior sub-regions of the deltoid during scapular-plane abduction, coronal-plane abduction and flexion [36]

		Scapular-plane abduction				Coronal-plane abduction				Flexion			
Muscle/muscle sub-region		Max	*θ*	Min	*θ*	Max	*θ*	Min	*θ*	Max	*θ*	Min	*θ*
Anterior deltoid	Anatomical	39.3	120.0	2.1	2.5	30.2	120.0	2.0	2.5	40.0	120.0	11.6	2.5
	RSA	38.6	97.5	7.4	2.5	35.8	90.0	15.6	2.5	36.0	75.0	25.9	2.5
Middle deltoid	Anatomical	33.1	120.0	6.7	2.5	29.1	86.3	8.3	2.5	12.2	120.0	0.0	2.5
	RSA	42.9	82.5	22.5	2.5	46.3	86.3	30.2	2.5	27.0	120.0	14.2	2.5
Posterior deltoid	Anatomical	−14.9	34.0	3.0	120.0	−15.9	5.0	2.0	120.0	−33.0	30.0	−16.3	120.0
	RSA	−12.4	2.5	5.2	120.0	14.1	120.0	1.3	2.5	−17.6	27.5	−13.1	108.8

Moment arm magnitudes (mm) are given, as well as the joint angles at which they occur. Data are displayed for the natural anatomical shoulder and the shoulder after reverse total shoulder arthroplasty (RSA). A positive value indicates an elevator, whereas a negative value indicates a depressor

### Surgical approach

Surgical approach is an important factor in RSA, as it is known to greatly influence post-operative muscle function and therefore clinical outcome [42]. The two most common techniques used are the delto-pectoral approach and the antero-superior deltoid splitting. The delto-pectoral approach minimises damage to the deltoid, which may improve post-operative elevation function and range of motion. In addition, it is thought that this approach allows for greater glenoid exposure therefore improving intra-operative implant positioning. Ultimately, this may influence the surgeon’s judgment of factors such as inferior glenoid tilt and glenoid version, which may contribute to scapular notching and affect post-operative range of motion and joint stability [43]. Unfortunately the delto-pectoral approach is known to compromise the subscapularis and potentially increase risk of joint dislocation [43, 44]. The subscapularis is an important stabiliser of the shoulder joint, opposing the action of the teres minor, and thereby generating compressive joint force by the resultant transverse-plane force couple. Damage to the subscapularis may disrupt this stabilising mechanism, resulting in joint instability.

The antero-superior deltoid splitting approach preserves the integrity of the subscapularis, and therefore may result in better post-operative joint stability. Some reports suggest that this technique yields poor exposure of the glenohumeral joint and thus may lead to a tendency of the surgeon to inadvertently tilt the glenoid base plate superiorly, resulting in intra-operative impingement on the scapula by the proximal humerus [45]. Other reports suggest a tendency for the surgeon to unintentionally resect more of the proximal humerus, which must then compensated for with a larger humeral polyethylene insert in order to obtain stable reduction [46].

### Scapular notching and adduction deficit

**Fig. 3:**
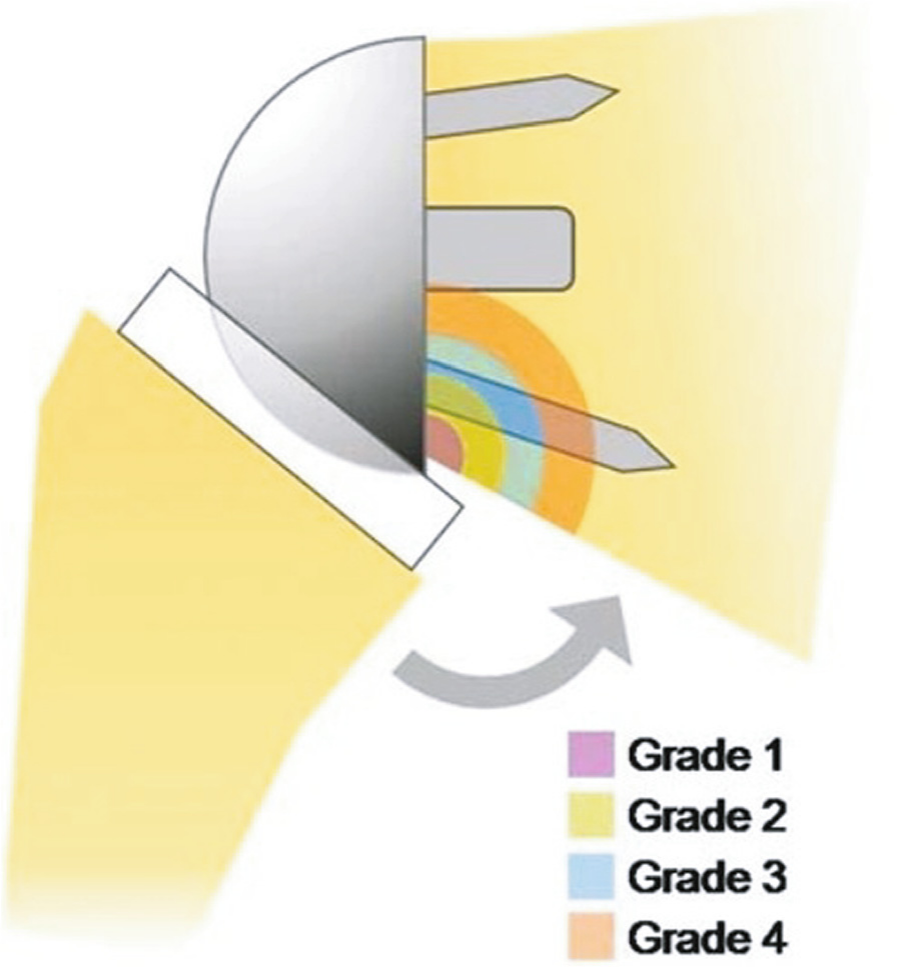
Nerot Sirveaux’s classification of inferior scapular notching

**Fig. 4:**
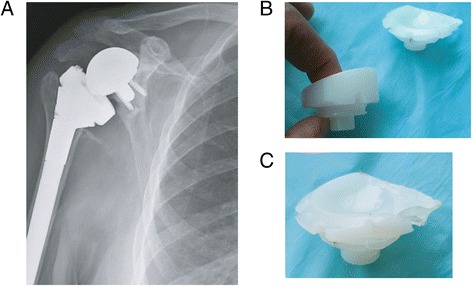
Grade 4 notching with osteolysis resulting in glenoid loosening (**a**), the original polyethylene humeral liner component (**b**) and the same humeral liner component retrieved after notching and glenoid loosening (**c**)

Medialisation of the reverse prosthetic glenohumeral joint may lead to scapular impingement or ‘notching’. Scapular notching refers to the gradual erosion of the scapular neck inferior to the peg or geometric centre of the glenoid implant. This is considered to be a result of direct mechanical abutment of the polyethylene humeral tray against the scapular neck as the arm is placed in adduction. Scapular notching, which has been reported in up to 80 % of cases [16, 47], is frequently graded using Sirveaux’s classification [20] (Fig. 3). Of particular concern is grade 4 notching (up to the inferior screw and glenoid peg) which may result in glenoid loosening (Fig. 4). Ultimately, scapular notching resulting in adduction deficit has the potential to generate polyethylene wear debris which can stimulate osteolysis [48]. This has prompted significant implant design modification and surgical technique review.

### Glenosphere lateralisation

Minimisation of scapular notching has been achieved using a more lateralised glenosphere offset and projecting the joint centre of rotation laterally relative to the glenoid face (Fig. 2c). This is the rationale behind the design of Reverse Shoulder Prosthesis (RSP, DJO Surgical, Austin, Texas, USA), which offers increased glenosphere proportions with up to 10 mm of lateral offset. While lateralised implant designs have resulted in lower incidence of notching [18, 49], they have also been associated with higher rates of base plate failure. This is due to the fact that a lateralised glenosphere creates a lever between the joint centre of rotation and the glenoid-baseplate interface, presenting risk of glenosphere failure due to torque transmitted from the upper limb directly to the glenoid baseplate.

Bony increased offset reverse shoulder arthroplasty (BIO RSA) is a technique modification used in conjunction with the Aequalis Reversed Shoulder System (Tornier Inc., Houston, Texas, USA). A discoid piece of bone autograft, generally harvested from the excised humeral head, is introduced between the native glenoid and the glenosphere and secured with use of a specific glenoid base plate (metaglene) incorporating a lengthened central peg [50]. The BIO RSA technique maintains the centre of rotation at the glenoid face but lateralises the entire construct. As a consequence, the torque loads transmitted to the baseplate are potentially lower than those in the lateralised RSP or Arrow designs. The BIO RSA technique may prove useful for primary RSA with marked glenoid wear or in revision RSA with resulting glenoid bone loss.

### Neck-shaft angle and effective angle of inclination

Changing the humeral neck-shaft angle from the Grammont standard 155° in the Delta III, to 145° in the Equinoxe (Exactech, Inc., Gainesville, Florida, USA) or to 135° in the RSP, SMR and Comprehensive (Biomet, Warsaw, Indiana), may confer biomechanical advantage and reduce adduction deficit [50], as the joint centre of rotation is shifted inferiorly.

Implants such as the Zimmer trabecular metal reverse shoulder system (Zimmer, Warsaw, Indiana) have a 5–10° wedged humeral polyethylene insert which can alter the effective angle of inclination; however, a thicker polyethylene liner can produce greater wear debris in the event of impingement and notching.

### The eccentric glenosphere

Glenosphere eccentricity may be achieved by shifting the glenosphere centre of rotation without altering the position of the base plate. The SMR, Aequalis, Delta III, Arrow and several other designs offer an eccentric glenosphere option. Clinical studies, mathematical modelling and sawbone-based experiments suggest that inferior eccentricity of the glenosphere may mitigate adduction impingement by shifting the glenohumeral joint centre of rotation inferiorly [51–53]. Eccentricity may also be employed anteriorly or posteriorly in the event of impingement or instability.

In a cadaveric study, Nyffeler and colleagues demonstrated that by placing the metaglene base plate on the inferior glenoid margin rather than in the centre of the glenoid, a glenosphere overhang was created that made impingement far less likely due to the increased space created between the humeral tray and the scapula [54]. This finding was confirmed in a retrospective clinical series by Simovitch [55] and corroborated in later computer modelling studies which concluded that shifting the metaglene inferiorly was the single most significant factor in mitigating impingement of the scapula [56, 57]. However, Nyffeler highlighted that this inferior shift may be complicated by insufficient distal bone stock in which to secure the obliquely oriented locking screw inferior to the central peg. A potential solution may be seen in the Affinis Inverse which has an additional horizontal peg rather than an inferior oblique screw.

### Inferior angulation of the glenosphere

**Fig. 5:**
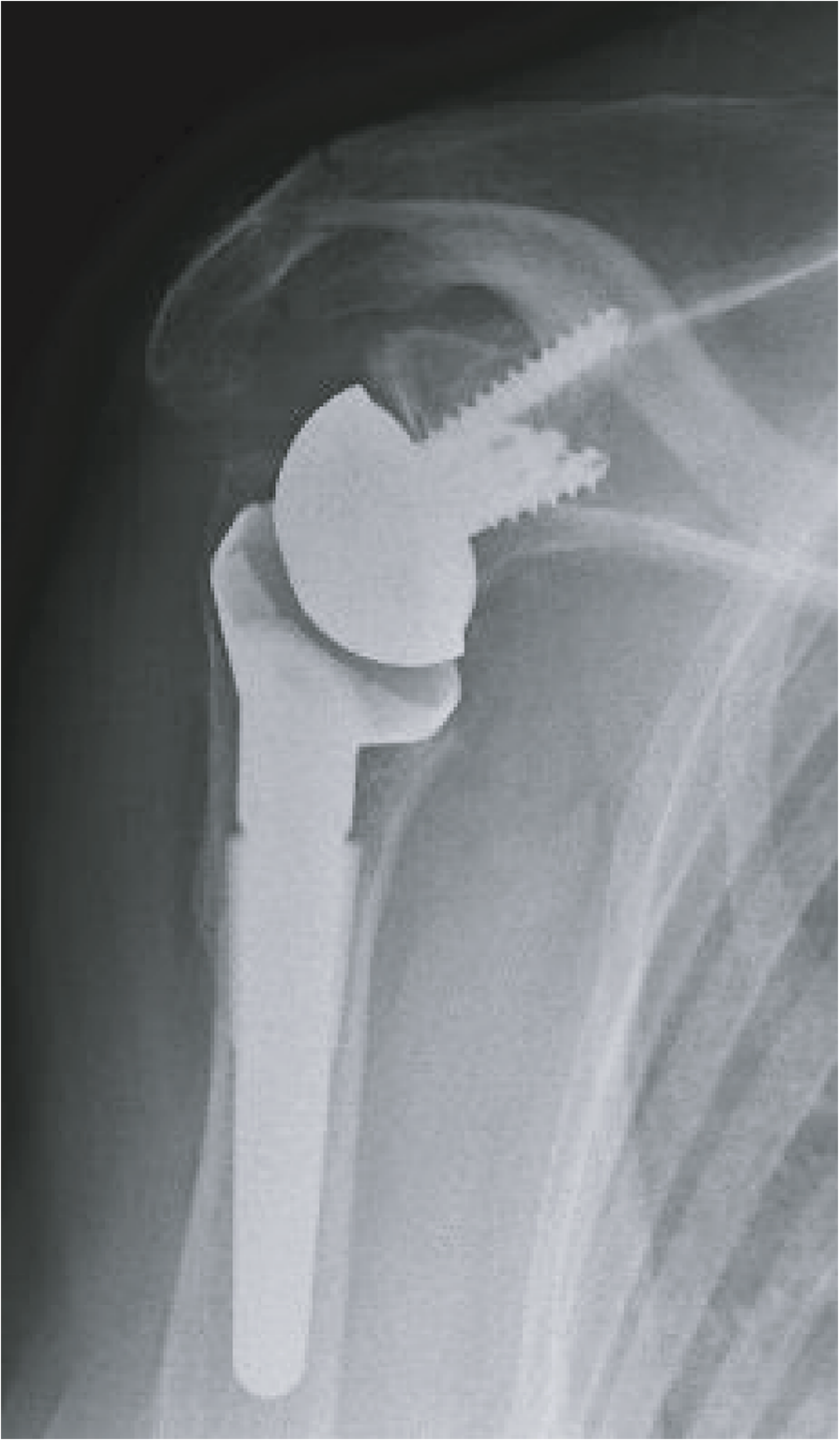
Inferior angulation of the glenoid component to mitigate scapular notching

Inferior angulation of the metaglene is an alternative technique that may reduce scapular notching [57] (Fig. 5). Suggested by Sirveaux et al. [20], this method is combined with inferior placement of the metaglene and was a response to poor clinical outcome in cases of superior glenoid wear (Favard classification 2 and 3). Cadaveric and computer model studies have suggested a potential benefit [54, 56], but in neither investigation was inferior angulation the most important factor in mitigating notching. In a prospective randomised clinical trial involving 42 Aequalis implants followed for a minimum of 1 year, 10° of inferior tilt actually provided no protection against notching as compared to neutral glenoid reaming [58]. A retrospective cohort trial reviewing 71 Delta III implants again revealed no mechanical benefit [59]. Inferior inclination has the disadvantage of requiring additional reaming in order to generate tilt, resulting in loss of glenoid bone stock and further medialisation of the joint centre of rotation. Inferior inclination combined with a lateralised design will ultimately reduce the amount of lateralisation obtained. The effect of inferior tilt may thus show a design-dependent effect, which is also true of the contact forces at the baseplate-bone interface. Inferiorly shifted eccentric glenospheres may generate an uneven distribution of glenohumeral joint force across the metaglene when placed in an inferior tilt. This may produce a ‘rocking horse’ effect at the glenoid, not seen in concentric implants. While this has only been demonstrated in a computer model to date [60], it is another example of important consequences of design variations within the family of reversed anatomy prosthetic joint components.

### Bearing surfaces

Traditional articular surfaces in the reverse shoulder prostheses have a metal glenosphere and a polyethylene cup insert over the humerus. However, others offer the reverse anatomy components with a polyethylene glenosphere and a metal cup insert over the humerus. Examples of this bearing surface style are the Affinis Inverse (Mathys, Bettiach, Switzerland) and the 40- and 44-mm glenospheres of the Shoulder Modular Replacement SMR (Lima Corporate, San Daniele del Friuli, Italy). This has the theoretical advantage of minimising polyethylene debris if impingement occurs; however, this remains to be proven in long-term clinical studies.

### External rotation deficit

Prosthetic reverse shoulder components were developed to function in rotator cuff-deficient shoulders without the typical stabilising transverse-plane force couple produced by the simultaneous activity of the subscapularis, infraspinatus and teres minor. While their non-anatomical constructs shift the joint centre of rotation medially and inferiorly to recruit more fibres of the deltoid during abduction and flexion [36], biomechanical studies demonstrate that reverse prosthetic designs may shorten the external rotation moment arms of the teres minor and posterior sub-region of the deltoid, thereby reducing external rotation capacity [41]. In a multicenter study, it was shown that 27 % of patients had lost some external rotation compared with their pre-operative state, while 13 % had negative or no external rotation [61]. In a retrospective review of 191 replacements, a mixture of Delta III and Aequalis implants, Wall et al. found no statistical improvement in external rotation at a minimum of 2 years follow-up. When assessed with their arm at their side, the study population had an average of 6° of external rotation, down from 8° pre-operatively [9]. In contrast, elevation is typically improved in RSA by recruitment of the deltoid; in the same study, elevation increased from an average of 86° to 137°. Clinical studies concur that fatty atrophy of the teres minor in RSA results in even greater loss of external rotation movement and poorer clinical outcome scores [9, 16, 62]. In such cases, latissimus dorsi tendon transfer may be used to restore external rotation function [63].

There is some evidence that lateralised designs may maximise the capacity of rotation movements by maintaining tension in any remaining rotator cuff muscles [64]. This lateralisation has typically been achieved at the glenoid component. However, the Equinoxe and the Arrow have a lateralised centre of rotation not as a result of the glenoid component, but rather a lateralised intra-medullary axis for the humeral component. In this configuration, the polyethylene cup sits on top of the humeral stem in a lateralised position [65, 66]. Hamilton et al. suggest that one should consider RSA components with one of three design philosophies: medialised glenoid and medialised humerus (MGHM), lateralised glenoid and medialised humerus (LGMH, e.g. RSA), or medialised glenoid and lateralised humerus (MGLH, e.g. Equinoxe) [66]. Using a computer model, they suggested a design-dependent increase in moment arms of the external rotators and therefore the potential of a corresponding increase in range of movement for the patient. While this has not been proven clinically, the disability caused by limitation of external rotation at the shoulder is well recognised and an important impairment in performing activities of daily living [67]. Rotation appears to be of particular practical importance during abduction or elevation away from the body. Therefore, reports assessing shoulder axial rotation capacity with the elbow positioned by the side should be interpreted with caution. Sirveaux et al. reported that external rotation assessed with the arm at the side showed no statistical improvement in their 80 cases; however, when measured with the shoulder in 90° of abduction, a significant improvement was demonstrated post-operatively [20]. Their suggested explanation was recruitment of the deltoid with abduction, which in turn aided external rotation.

Humeral version may also play a role in axial rotation. Gulotta et al., in a cadaveric model, investigated the effect of humeral version on muscle recruitment and impingement-free arc of movement [68]. They could not demonstrate any meaningful change in biomechanical muscle force generated in teres minor but found that with increased humeral retroversion, there was an increased range of impingement-free external rotation; however, this was at the expense of internal rotation [68]. Humeral version may simply alter the arc in which the available rotation occurs. While increasing retroversion may delay impingement during external rotation, it may mean that impingement occurs earlier in internal rotation [69].

A further factor that may contribute to loss of external rotation is inadvertent damage to the suprascapular nerve from malpositioning of baseplate metaglene screws [70, 71]. Penetration of the suprascapular nerve may affect infraspinatus function and therefore external rotation function. While the numbers of screws used in baseplate fixation varies with implant design, from two in the SMR, to four in the Delta III design, and six in the Equinoxe, the screws that present the greatest risk of nerve damage are the superior and, if present, the posterior screws. This is not likely to be a common factor in the determination of external rotation when compared to the pre-operative state of teres minor, the extent of construct medialisation or humeral version, but it is within the control of the operating surgeon and is another example of a design-dependent factor that may influence clinical outcome.

### Dislocation

Dislocation was found to be the most common complication of RSA by Wall et al. when they retrospectively reviewed 199 procedures associated with a variety of indications. They identified fifteen dislocations, a prevalence of 7.5 % for their study population [9]. It was also shown that revision procedures present higher risk of dislocation [9]. When reviewing results of RSA for failed fracture hemiarthroplasty, Levy et al. describe dislocation in 5 of 29 patients, 3 of whom experienced recurrent dislocation [72]. Thus, the risk of dislocation may be dependent on the original indication for surgery [73].

Reconstruction of the subscapularis affects the risk of dislocation in the reverse shoulder [15]. Edwards et al., in a review of 138 consecutive Aequalis implants, identified seven patients who suffered dislocation within 2 months of their operation (5.1 %). All had been identified as having an irreparable tear to the subscapularis at the time of operation. Relative dislocation incidence in those without a subscapularis repair was just 1.9 % [73]. The pre-operative diagnosis of a subscapularis repair was also strongly associated with dislocation incidence, perhaps reflecting the difficulty incurred in repairing the subscapularis.

Humeral version may also play a role in increasing joint stability post-operatively. Using a mechanical model, Favre et al. found that increasing glenoid retroversion produced glenohumeral instability, whereas increasing anteversion of the humerus produced greater stability by joint compression [74]. They concluded that glenoid retroversion of more than 10° should be avoided and that humeral version should be neutral or slightly anteverted due to the negative effect on external rotation range of motion. Inferior glenoid inclination has also been suggested as mechanism to reduce dislocation. In a retrospective study, Randelli et al. describe a cohort of 33 patients all of whom underwent RSA with a Delta Xtend reverse prosthesis with varying degrees of glenoid tilt. Two atraumatic dislocations occurred (6 %) within the first 2 months. One had a positive inclination of 6.9° and the other a negative of 2.4°. All stable implants had an average negative inclination of 9.4° [75]. While these results suggest a dislocation protection effect with inferior inclination, further prospective studies are required to explore this association and its effect on glenohumeral joint compression.

## Conclusion

RSA is an evolving technique. Indications for surgery, operative technique, implant design and the avoidance of complication are dependent on fundamental principles of biomechanics. Surgical technique and prosthesis design can have a significant influence on clinical outcome of RSA and implant longevity. Scapular notching and external rotation deficit are predominantly influenced by joint centre of rotation position and post-operative muscle leverage, respectively. These factors can vary substantially with implant design. While short-term results of RSA remain positive, especially in cases of difficult to treat pathologies such as cuff tear arthropathy, uniformly satisfactory long-term results are yet to be achieved. Scope for future research and prosthetic design development lie in a better understanding of the influence of optimum bearing surfaces, glenoid diameters, implant version, inclination and offset and their effect on muscle and joint function, since these design parameters are highly relevant to clinical outcome.
